# An Evaluation of Food Safety Performance in Wineries

**DOI:** 10.3390/foods11091249

**Published:** 2022-04-26

**Authors:** Jesús López-Santiago, Ana Isabel García García, María Teresa Gómez-Villarino

**Affiliations:** Agroforestry Engineering Department, School of Agricultural, Food and Biosystems Engineering, Universidad Politécnica de Madrid, 28040 Madrid, Spain; ai.garcia@upm.es (A.I.G.G.); teresa.gomez.villarino@upm.es (M.T.G.-V.)

**Keywords:** beverages, HACCP, food hazards, food safety, wine, wineries

## Abstract

Wine production has food safety hazards. A Hazard Analysis Critical Control Point (HACCP) system makes it possible to identify, evaluate, and control significant food safety hazards throughout the wine production process. The Prerequisites Programs (PPRs) and HACCP performance in Protected Denomination of Origin “Vinos de Madrid” wineries were analyzed. Winery performances were evaluated for every critical control point (CCPs) in each winemaking process stage, including their implementation of PPR and HACCP principles. This study was developed through a survey of 55 questions divided into 11 sections, and it was conducted on a sample of 21 wineries. The results revealed that the CCPs worst performance level are for the control of metals (Cd, Pb, As) in grapes and fungicides or pesticide control in the harvest reception. A total of 91.5% of the wineries had implemented a prerequisites program (PPRs), regardless of their annual wine production. However, there was variability in the type of prerequisite plans, training, level of knowledge of operators, and annual budget allocation. Three out of four wineries had an HACCP, although corrective action procedures and verification procedures had the lowest and the worst HACCP practical implementation. The significant barriers for HACCP performance in wineries are linked with a lack of food safety staff training, low involvement of all staff in food safety tasks, and poor application of CCP chemical and microbiologic control methods.

## 1. Introduction

Wine is an alcoholic beverage that results from the fermentation of grapes. The winemaking process follows appropriate steps along with the addition of certain additives, such as sulfur dioxide, tartaric acid, or egg albumin [1]. The wines are manufactured using a common generic process but with some variations depending on the type of wine that is being produced. In the European Union, these oenological practices are regulated by EC Regulations n° 423/2008, n° 479/2008, and n° 606/2009 [2,3,4].

The process of making red wine begins when grapes are harvested and transported to the winery. Grapes are crushed and stemmed, obtaining a semi-liquid composed of skins, seeds, pulp, musts, and scrapes. Subsequently, it receives corrections such as the addition of sulfur dioxide to obtain an antioxidant and an antiseptic effect. All these additives are regulated in the European Union by EC Regulations n° 1333/2008, n° 1129/2011, and n° 1130/2011 [5,6,7].

Alcoholic fermentation takes place in large vats, and it is triggered by yeasts when sugars are converted in ethanol [8]. Maceration and fermentation processes occur simultaneously [9,10]. The liquid part of the grape is also called must-wine. Must-wine is transformed by fermentation. At the same time, must-wine exchanges components with the solid parts of the grapes. The extraction of anthocyanins from the skins is of major interest because they are responsible for providing the final red color of wines [11,12,13]. Alcoholic fermentation takes from 10 to 20 days, depending on the temperature [14].

Vat is drawing off and the malolactic fermentation starts to transform malic acid into lactic acid by lactic bacteria [15]. Malolactic fermentation achieves the lasting stabilization of the wine, preventing the development of other types of bacteria [16]. Particles, such as yeasts, solids, or other organic matter, are decanted and deposited at the bottom of the vat. All these particles are known as “wine waste”.

Racking avoids any contact between the wine and the “wine waste”, which can cause losses in the organoleptic properties of the wine. Suspended particles are removed by adding clarifying or colloidal substances, such as egg albumin, gelatin, or alginate [17,18,19].

Wine is filtered through a porous material to retain possible solid particles that remain in the liquid phase eliminating turbid or precipitated wines [20].

Wine is stabilized chemically and biologically by cold keeping wine stable in the long term [17]. This stage inhibits microbial growth, and it avoids oxidative reactions. Finally, wine is bottled to protect it from external agents that deteriorate it.

During all of the above processes, the characteristic flavor and aroma of wines are formed [21,22]. However, grapes, must, and wines are susceptible to several safety hazards. These food safety risks include physical (metal parts, glass, insects), chemical (pesticide residues, heavy metal residues, urea), and microbiological (pathogens) hazards affecting the health of consumers [23].

Hazards that appear in wine production may come from the environment, processing equipment, and workers of the winery. Nevertheless, the two common causes of major food safety incidents are those related to undeclared allergens and cross-contamination [24]. Hygienic conditions and practices are required to prevent the introduction of hazardous agents. In winemaking, the main hazardous agents are the increase in the microbiological load or the accumulation of residues and other chemical and/or physical agents produced directly and indirectly.

According to European food law, “Food shall be deemed to be unsafe if it is injurious to health or unfit for human consumption” [25]. Food security occurs when “all people have permanent physical, social, and economic access to safe, nutritious food in sufficient quantity to meet their nutritional requirements and food preferences, and thus be able to lead an active and healthy life” [26].

This concept has been growing in importance over the last decades, extending not only to food but also to all links in the food chain, from primary production through the processes of transformation, manufacturing, handling, and packaging to sale to consumers and the preparation of food by the final consumer.

In this context, a critical control point (CCP) “is a point in a step or procedure at which a control should be applied for the prevention or elimination of a hazard or reduction to an acceptable level” [24]. A critical control point hazard analysis (HACCP) makes it possible to identify, evaluate, and control significant CCPs throughout the food production process. Once these possible hazards have been identified, whether they constitute a critical control point (CCP) is assessed, and then the reference limits or critical limits are established by applying preventive measures that prevent non-conformities or deviations from these established limits [27,28,29].

Hazard Analysis Critical Control Point (HACCP) systems are defined as preventive programs focused on the production of safe food products [23]. The HACCP system emerged in 1959 when the Pillsbury company together with the United States Army and the National Aeronautics and Space Administration (NASA) developed a program to produce safe food. It was considered that the most important diseases that could affect astronauts were those related to the food they would consume [27]. The Pillsbury company introduced HACCP as the system that could offer the highest food safety because it controlled the entire production process from the beginning of the processing chain. The HACCP program had only three principles in its origins, while it currently has seven. The National Academy of Sciences (NAS) [30] and the National Advisory Committee on Microbiological Criteria for Foods (NACMF) is responsible for this expansion and its subsequent adoption by the Codex Alimentarius in 1993.

In 2003, a global consensus was reached for the use of HACCP in the global food supply. The Codex Alimentarius Food Hygiene Committee of the World Health Organization issued the Hazard Analysis and Critical Control Points guidelines for International Trade [31,32]. Soon after, the European Union, Canada, Australia, and Japan issued regulations requiring food businesses to develop and to implement food safety plans based on the NACMCF and the Codex Alimentarius frameworks [33].

Nowadays, the HACCP method is an internationally recognized systematic approach to food security that seeks to comply with the development and the realization of effective food safety practices [34]. It is based on seven principles that identify and control food safety hazards (Table 1) [34,35]. In addition, HACCP ensures that appropriate corrective actions are taken, where necessary, and that a registration system is available for documentation [36]. Under these principles, if any deviation occurs, it indicates that control has been lost. In that case, it is of paramount importance taking appropriate steps to restore control and to ensure that potentially dangerous products do not reach the consumer [37].

An HACCP program is required to be built on a solid foundation of previous programs called prerequisite programs (PPRs) [38]. In accordance with what is described in the Codex Alimentarius [31,32], PPRs are based on the general principles of food hygiene [39]. Thus, PPRs provide basic environmental and operating conditions that are necessary to produce safe and healthy food. They address issues related to the cleaning and the disinfection of facilities and equipment, the supply and the use of supply water, pest prevention and control, staff handling practices and knowledge of food safety, and the identification and the location of food produced and marketed [29,40]; PPRs are built with different types of programs that develop each issue.

While the HACCP focuses on the dangers that depend solely on the production process of the wines, PPRs seek to eliminate all those dangers that depend on the work environment in the winery. The types of PPRs depend on the winery in which the HACCP is going to be implemented, and they have to be adapted in a specific way.

The application of HACCP has advantages, but it also has certain drawbacks. The main advantages from its application are guaranteeing food safety, allowing the control of each of the phases of the food chain that intervenes in the elaboration of a product, facilitating the supervision of the system by competent authorities, and its application to all food companies, regardless of their size or activity.

However, HACCP presents certain drawbacks, such as the need for: specific technical and material resources that are not always available to the industry; the training and the commitment of industry employees; methods for determining difficult critical control points [41]; and continuous evaluation and analysis of data [38].

Consequently, the aim of this study is to evaluate PPRs and HACCP implementation performance in wineries and the identification of the main barriers that hamper an optimal implementation.

## 2. Materials and Methods

### 2.1. Study Design

The performance of an HACCP program was assessed in a Protected Designation of Origin (PDO) “Vinos de Madrid” wineries sample. A survey was conducted in this sample in the last quarter of 2021. The collected data were analyzed with statistical methods using the SPSS for Windows software (IBM Corp. Released 2020. IBM SPSS Statistics for Windows, Version 27.0. Armonk, NY, USA: IBM Corp.). Frequencies and central position values were calculated for all variables. A Spearman correlation coefficient (*ρ*) for nonparametric measure of rank correlation with a significance level of *p* < 0.01, Kruskal–Wallis non-parametric test and contingency table were carried out. 

### 2.2. Sample Selection

Protected Designation of Origin “Vinos de Madrid” has a total population of 51 wineries. They produce 78% percent of the total wine production of the Madrid Region [42]. The sampling method was the non-probabilistic method. The sample selection was made using the researcher’s previous information, instead of random selection [43].

The food-safety staff of wineries were asked about their Prerequisite and HACCP programs performance by a survey.

The survey was sent by email to every winery of the sample. The e-mailing was made on three occasions. In addition, an attempt at telephone contact was made by each of the wineries in the sample. A total of 21 wineries answered the survey.

The response rate (RT) was the percentage of the eligible sample from which information was obtained [43]. The RT was 21/50 representing 42% of the sample. The non-response rate (NRT) was the ratio between the number of rejections contacted and the number of all units selected [43]. The NRT was 29/50, representing 58% of the sample. The NRT reduced the sample size to 21 wineries up to a population of 51.

### 2.3. Survey Preparation

The survey design was carried out using a structured questionnaire. Each question had a limited alternative answer. These types of questionnaires are used for conclusive and descriptive research [43]. The survey had a total of 55 questions divided into 11 sections.

The first section consisted of seven questions related to annual red wine production and PPRs implementation. The first question identified the winery size according to its annual red wine production. We considered five groups when preparing the first question. This group distribution covered all possible cases of responses according to the PDO “Vinos de Madrid” wineries annual red wine production [44]. The other six questions were focused on PPRs application. These questions asked about: how PPRs were drafted, communicated, and known by the winery staff; how PPRs application evidence and compliance were generated as updated records; and how PPRs are financed.

Sections two to ten were related to evaluating HACCP Principle 1 Perform a Hazard Analysis and HACCP Principle 2 Determine Critical Control Points (CCPs). These 9 sections contained a total of 37 items. These 37 items were prepared using the results obtained in several scientific and technical studies [23,45,46,47,48], and they provided qualitative information about the analysis and the control over the different hazards and critical control points (CCPs) in each stage of the red wine production process.

Each of the 37 items was assigned a qualitative scale “Never”, “Hardly ever”, “Usually”, and “Always”; it was coded using a Likert scale [43]; and a quantitative variable was assigned to each item, respectively. Although the recommended Likert scale is five categories, we truncated the scale to four categories to eliminate the “neutral” option in a forced choice survey [49,50,51]. The numerical scale correspondence was “Never” with zero (0), “Hardly ever” with one (1), and “Usually” with two (2), and “Always” with three (3). A high variability in a quantitative variable was considered when its interquartile range value was equal or greater than two.

Section 11 contained 11 questions about the practical implementation of principles 3 to 7 on which the HACCP was based. The HACCP Guidelines were used for question preparation [29,52]. Eight items were dichotomous, and three items were multiple choice.

Principle 3 Setting Critical Limits was evaluated with a dichotomous question Yes/No. However, an affirmative option introduced the assessment of whether the winery only focused on the application of the legislation or if the winery also included the technical recommendations made by professional entities of viticulture.

Principle 4 Establishing a Surveillance System was evaluated with three questions. A dichotomous question about a surveillance system for CCPs establishment, a multiple-choice question about the CCPs implemented verification methods, and a dichotomous question about the implementation of a hazard monitoring actions program.

Principle 5 Establishing Corrective Measures was evaluated with one dichotomous question that asked about establishment of procedures for corrective measures.

Principle 6 Establish Verification Procedures was evaluated with four questions. A dichotomous question about the CCPs system establishment of verification procedures, a dichotomous question about the control frequency of each critical control point and the person responsible for carrying it out, a multiple-choice question about staff involved in verifying the effectiveness of the CCPS system, and a dichotomous question about conducting an annual HACCP internal audit that introduced the periodicity factor in the affirmative option.

Principle 7 Establish a System of Registration and Documentation was evaluated with two questions: a dichotomous question about HACCP registration and documentation implementation that introduced the factor of updating and the periodicity with which the documentary review was carried out; and a multiple-choice question about the type of documents included in the winery’s HACCP.

The study tested three research hypotheses: Hypothesis 1 (H1) the effectiveness of the PPRs performance is different in each winery; Hypothesis 2 (H2) CCPs control performance is different for each of them; and Hypothesis 3 (H3) HACCP principles have different levels of implementation in wineries.

## 3. Results

The wineries were distributed according to their annual wine production. Four groups were considered instead of five, as we did not get any response for the group with an annual wine production greater than 500,000 L/year. Thus, annual wine production was recorded as follows: 47.6% of the wineries had an annual wine production between 25,001 and 100,000 L/year; 23.8% of the wineries had an annual wine production between 100,001 and 250,000 L/year; 23.8% of the wineries had an annual wine production of up to 25,000 L/year; and 4.8% of the wineries had an annual wine production between 250,001 and 500,000 L/year.

### 3.1. Prerequisites (PPRs) Implementation

In total, 91.5% of wineries had implemented a prerequisites program, regardless of their annual wine production; 9.5% of wineries had not implemented a prerequisites program.

**Figure 1:**
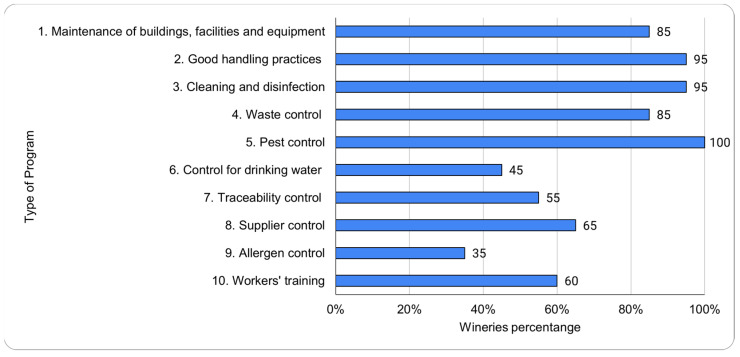
Percentage of wineries that have implemented each type of program included in a standard prerequisites program (PPRs).

The percentage of implementation of each type of program that made up a standard PPRs [29] in the wineries is showed in Figure 1.

A pest control program was implemented in all the wineries; 95% of wineries had implemented a cleaning and disinfection program and a good handling practices program; and 85% of wineries had a maintenance of buildings, facilities and equipment program and a waste control program. However, a traceability control program (55%), supplier control program (65%), workers’ training program (60%), and control for drinking water program (45%) were implemented in nearly one out of two wineries. Finally, the allergen control program (35%) existed in only one out of three wineries.

Table 2 shows the results regarding the percentage of workers that have received training in good manufacturing practices in winemaking (GMP) and the percentage of workers with knowledge about the PPRs by type of winery, according to their annual wine production, and by the total of the wineries.

A total of 81% of wineries had at least 50% of workers that had received training on good manufacturing practices in winemaking (GMP), the results obtained indicate that 81% of wineries had at least 50% of the staff trained in GMP.

Regarding their annual wine production, the wineries that exceed 100,000 L/year were those with the highest percentage of workers that had received GMP training.

In total, 95% of wineries indicated that more than 50% of workers had knowledge about their prerequisite program, while 62% of wineries had all their workers knowing their PPRs, and 38% of the wineries had more than 50% of their workers knowing their PPRs. In contrast, 20% of wineries with annual wine production up to 25,000 L/year hadn´t any workers with GMP training received.

The results obtained regarding the existence of an annual economic endowment through a detailed annual budget for the development of the prerequisite program are shown in Table 3.

A total of 62% of the wineries were making plans according to their needs without having a specific annual budget beforehand. This fact occurred in 82% of wineries between 25,001 and 100,000 L/year and in 60% of wineries between 100,001 and 250,000 L/year.

The existence of a specific annual budget occurred mainly in the largest wineries: 40% of the wineries between 100,001 and 250,000 L/year and in 100% of the wineries between 250,001 and 500,000 L/year, while 60% of wineries with up to 25,000 L/year did not have any annual budget.

### 3.2. Critical Control Point Hazard Analysis (HACCP) Implementation

A total of 76.2% of wineries had implemented a HACCP. One out four wineries had no HACCP. This occurred in 30% of wineries with an annual production between 25,001 and 100,000 L/year, in 20% of wineries with an annual production between 250,001 and 500,000 L/year, and 20% of wineries with production up to 25,000 L/year.

#### 3.2.1. Performance in Principles One and Two: Critical Control Points

The results of CCPs control performance are shown in Table 4. This contingency table [43] is formed by the medians of each of the categorical variables associated to each CCP analyzed. Variables appear in rows and their median values appear in columns. In addition, the last right column shows which variables have a high variability.

Five groups were determined considering the median value of each variable and the existence of high variability measured by the interquartile range.

Group I is formed by variables with a median equal to zero, corresponding to the fact that the CCPs linked to “Never” were not controlled. Additionally, variables have a high variability. Thus, VAR2.3 and VAR4.1 belong to this group, and they are shown with a red cell and “Y” in Table 4.

Group II is formed by variables with a median equal to one, corresponding to the fact that the CCPs linked to “Hardly ever” were controlled. Additionally, variables have a high variability. Thus, VAR2.1, VAR8.1, and VAR9.3 belong to this group, and they are shown with an orange cell and “Y” in Table 4.

Group I and Group II represent the Critical Control Points (CCPs) that are worst controlled in wineries, and they therefore pose a high risk for the safety of the final product.

**Figure 2:**
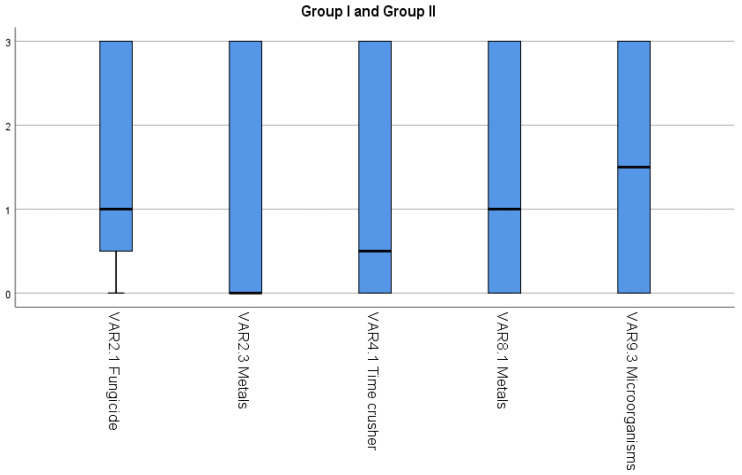
Median and range interquartile for variables included in Group I and Group II.

Figure 2 shows that absence of contamination control by metals (cadmium, lead, arsenic) in grapes at the harvest reception and time control that must be kept in the crusher are the critical points not controlled in 50% of wineries. Critical points that are poorly controlled in 50% of wineries include: control over fungicide residues and/or pesticides in grapes at the harvest reception in the winery, control of the concentration limits of metals (traces of As, Cu, Pb) in the wine during cold stabilization, and controlling the absence of microorganisms in the bottling line at the bottling and labelling stage. A strong positive correlation was found between the presence of fungicide residues and/or pesticides in grapes and contamination by metals (cadmium, lead, arsenic) in grapes (*ρ* = 0.850). 

Group III is formed by variables with a median equal to two, corresponding to the fact that the CCPs linked to “Usually” were controlled. Additionally, variables have a high variability. Thus, VAR5.1, VAR6.1, VAR7.4, and VAR9.1 belong to this group, and they are shown with a light green cell and “Y” in Table 4.

Group IV is formed by variables with a median equal to three, corresponding to the fact that the CCPs linked to “Always” were controlled. Additionally, variables have a high variability. Thus, VAR1.1, VAR1.3, VAR6.9, VAR7.3 and VAR9.4 belong to this group, and they are shown with a dark green cell and “Y” on Table 4.

**Figure 3:**
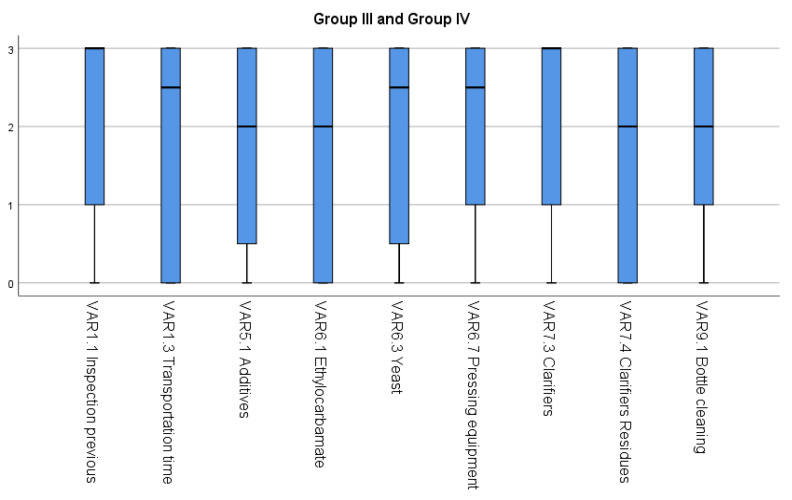
Median and range interquartile for variables included in Group III and Group IV.

Group III and Group IV represent the Critical Control Points (CCPs) that are well controlled in at least 50% of the wineries, however, there is a high variability that shows a lack of control in the other 50%. Figure 3 shows these critical control points: grape inspection during the previous harvest in vineyards and the transportation time of the harvest from vineyards to wineries; the safety and the purity control of the additives used in the sulphited and the vatted stage; control of the concentration of ethylocarbamate in fermented must; the purity and the safety control of yeasts for fermentation; control of the cleaning of pressing equipment after pressing; the purity and the safety control of agents used as clarifiers; control of residue from wine clarifiers, control of bottle cleaning procedures, and control of microorganisms in bottle caps. 

A strong positive correlation was found between the safety and the purity control of additives and the safety control of agents used as clarifiers (*ρ* = 0.929), the safety and the purity control of additives and the control of residue from wine clarifiers (*ρ* = 0.916), the purity and the safety of yeasts and the concentration of ethylocarbamate in fermented must (*ρ* = 0.831), the safety control of agents used as clarifiers (*ρ* = 0.929), and the control of residue from wine clarifiers (*ρ* = 0.916).

Group V is formed by variables with a median equal to three, corresponding to the fact that the CCPs linked to “Always” were controlled. These variables represent all Critical Control Points that are well controlled, and they are shown with a dark green cell in Table 4.

A Kruskal–Wallis test for independent samples in all groups defined on the null hypothesis that the distribution of the qualitative variable is the same between the different categories of annual wine production allowed for the consideration that the median and its variability is the same regardless of the annual wine production of the wineries. The result of this test accepted the null hypothesis for all the variables studied, except for VAR6.1

#### 3.2.2. Performance in Principles Three to Seven

The results of the practical implementation of HACCP Principles three to seven are reflected in Table 5, considering annual wine production of wineries. No data was received for wineries over 250,001 L/year.

Regarding Principle 3, Table 5 shows that 56.3% of wineries established the target levels and critical limits for each of the CCPs identified, following the mandatory regulations and/or professional technical recommendations. A total of 37.5% of wineries established target level and critical limits, but only for those in which there are applicable mandatory regulations. Only 6.2% of wineries did not set any target or limit for CCPs. The smallest wineries, producing up to 25,000 L/year, showed the best performance for this principle.

In total, 93.8% of wineries implemented a surveillance system of critical control points in accordance with Principle 4.

The sum of 68.7% of wineries had a written procedure for the establishment of the corrective measures to be applied in case of identifying deviations in each of the CCPs following Principle 5.

To verify the effectiveness of the HACCP system in compliance with Principle 6, 68.7% of wineries had a procedure.

The worst performance for Principles 5 and 6 was shown in wineries between 25,001 and 100,000 L/year. One out of two did not implement a procedure of corrective measures or a verification procedure.

With respect to Principle 7, 50% of wineries had a complete and periodically updated registration and documentation system; 43.8% of the wineries had written records and documents, but they were incomplete or not updated periodically.

The type of verification methods used by the wineries for the surveillance of CCPs were evaluated according to Principle 4: 93.8% of the wineries performed visual observation, 87.5% performed physical determinations (temperature, relative humidity, pH), 81.3% performed sensory assessment (smell, taste, aroma, texture), 81.3% performed chemical analyses, and 43.7% performed microbiological analyses. In addition, 81.3% of wineries had a written surveillance program that detailed the actions for hazards and their CCPs monitoring at each wine production stage.

Regarding the implementation of Principle 6, the profiles of professionals that were involved in the verification of the HACCP system were as follows: 100% oenologists, 53.8% managers, 46.2% of winery owners, 15.4% quality managers or similar, and 15.4% winery operators.

Table 6 shows the control frequency and the audit achievements in the wineries. These two elements serve to verify the practical implementation of Principle 6.

In total, 71.4% of the wineries detailed the control frequency of each CCP and the person(s) responsible for carrying it out.

A total of 35.7% of wineries did not carry out audits, 35.7% of wineries carried out annual audits, and 28.7% of wineries did audits but with a periodicity greater than a year.

**Figure 4:**
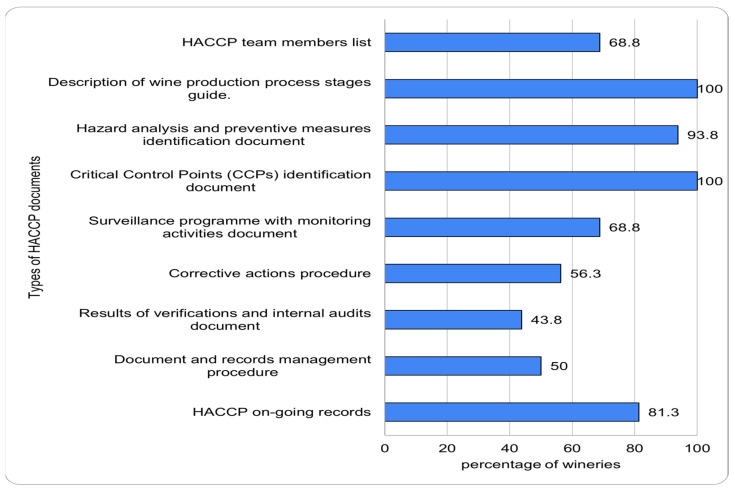
The percentage of total wineries that have included each type of document in their HACCP system.

Principle 7 is developed by 9 types of documents. The percentage of total wineries that have included each type of document in their HACCP system is shown in Figure 4. All wineries had a description of wine production process stages guide, 100% of wineries had a Critical Control Points (CCPs) identification document, 93.8% had a hazard analysis and preventive measures identification document, 81.3% had an HACCP on-going records, 88.8% had an HACCP team members list, 88.8% had a surveillance program with monitoring activities, 56.3% had a corrective measures procedure, 55% had a documents management system and a registration procedure, and 43.8% had results of verifications and internal audit documents.

## 4. Discussion

The correct implementation of prerequisites programs and a Hazard Analysis Critical Control Point (HACCP) system are essential to prevent illnesses and to ensure food safety for people. Although, the methodologies of practical development of both instruments for food and hygiene safety is widely spread and studied worldwide, it has been proven that there are companies that still do not manage to obtain the maximum effectiveness in food safety [53,54,55]. This study provides an overview of performance in the implementation of prerequisite programs and the HACCP in wineries in the Madrid Region

The study results prove Hypothesis 1. They show that the effectiveness of the PRRs performance is different in each winery. Although PPRs implementation is widespread in DPO “Vinos de Madrid “wineries, their practical deployment level through compliance with prerequisite plans (PPR) is different. A total of 95% of wineries have a PPR in place, regardless of their annual wine production level; although, their PPRs practical implementation are different in terms of the types of plans implemented, the operators’ levels of training and knowledge about them, and the annual budget allocated for PPRs execution.

Thus, one hundred percent of the wineries have a pest control plan, while only 35% carry out an allergen control plan. Most of the wineries have operators trained in good winemaking practices and prerequisites, but only one out of four have established annual PPRs budgets. Both factors, workers’ training levels and economic allocation appear as causes of the diversity in the level of deployment of PPRs.

It is recognized that without a good implementation of PPRs, it is difficult to correctly develop a HACCP.

The study reveals that Hypotheses 2 is true. The CCPs performance control is different for each of the 37 evaluated CCPs (see Table 4). A total of 22 CCPs are well-evaluated by the wineries. However, there are significant differences among another 15 CCPs depending on each winery. The worst control performance among wineries for CCPs appears related to chemical controls of metals traces, fungicides and pesticides in grapes or wine, biological controls of microorganisms in equipment, and operations stage controls as the remaining time of the must in crushers. Deficiently controlled CCPs assume that hazards such as the appearance of microorganisms, trace metals, fungicides, pesticides or other dangerous products in grapes or wine may occur [23,46,56].

Hypotheses 3 is proven. The results show HACCP principles have different implementation levels in wineries. The worst implemented HACCP principles are Principle 5 and Principle 6. Only 78.7% of the wineries had established a corrective measures procedure and a verification procedure.

Additionally, documentary and registration systems present great variability in their practical implementation in wineries. Thus, most have identification documents of critical control points and hazard analysis and determination of preventive measures, while less than half do not have the results of verifications and internal audits.

In total, 93.8% of wineries had established a CCP monitoring system, but microbiological testing methods were used by less than 50% of wineries. In addition, in almost half of the wineries this surveillance falls solely on the oenologist, without involving the rest of their workers.

## 5. Conclusions

This work demonstrates the need to continue improving in the technical implementation of the HACCP methodology and in training workers about food safety in wineries.

According to the barriers identified for the development and the implementation of HACCP by Vela & Fernández [38], the establishment of new CCPs surveillance formats involving the different professionals working in the wineries (enologist, manager, winery operators, quality managers) will improve performance in the CCPs surveillance, and by extension, the deployment level of the entire HACCP. Mojca Jevsnik stressed the importance of a motivated, satisfied, and qualified personnel to assure an efficient HACCP [57].

A good implementation of PPR and HACCP contributes to eliminating food safety risks that compromise people’s health. The first step forces the winery industry to improve the identification, analysis, and evaluation of hazards and CCPs. To do this, wineries must make improvements in their chemical and microbiological analysis laboratories. At the same time, they must invest in training and sensitization of all staff in matters aimed at methodological knowledge and a good development of PPRs and HACCP.

Leadership is essential to a good culture of safety [58]. Winery owners and managers must demonstrate a full commitment to the good functioning of food safety systems. This makes it easier for all workers to align with senior management to achieve the effectiveness of food safety systems.

Research on the analysis of training levels in food safety for each of the professionals’ profiles working in the wineries is convenient, likewise research that relates the level of commitment of workers classes according to the level of training acquired on PPRs and HACCP.

Following these steps will help not only the wine industry but also the health of customers.

## Figures and Tables

**Table 1:** Key principles of Critical Control Point Hazard Analysis (HACCP).

Number	Principle	Description
Principle 1	Perform a hazard analysis	Hazards should be identified and the associated risks that accompany them should be assessed at each stage of the production system and possible control measures should be described.
Principle 2	Determine Critical Control Points (CCPs)	Critical control points must be determined.
Principle 3	Set critical boundaries	A critical limit must be associated with each control measure to ensure that critical control points (CCPs) are under control.
Principle 4	Establish a surveillance system	A surveillance system should be implemented to ensure that CCPs are within critical limits and therefore under control
Principle 5	Establish corrective measures	Corrective measures to be taken when the surveillance system detects that a CCP is outside the control limits should be established
Principle 6	Establish verification procedures	Verification procedures should be established to confirm that the HACCP is functioning effectively and correctly.
Principle 7	Establish a system of registration and documentation	A system of record should be established on all procedures performed and the associated records.

**Table 2:** GMP workers training and PPRs workers knowledge by type of winery, according to their annual wine production and by the total of the wineries.

Wine Annual Production	Percentage of Wineries Over Total	GMP Workers Training (%)			PPRs Workers Knowledge (%)		
		All	More than 50%	None	All	More than 50%	None
up to 25,000 L/year	23.8	50	0	50	40	20	20
between 25,001 and 100,000 L/year	47.6	36	55	9	50	50	0
between 100,001 and 250,000 L/year	23.8	60	40	0	80	20	0
between 250,001 and 500,000 L/year	4.8	100	0	0	100	0	0
Percentage of total wineries	100	48	38	14	62	33	5

**Table 3:** Annual budget of existing PPRs by type of winery, according to their annual wine production and by the total of the wineries.

Annual Wine Production	Percentage of Wineries Over Total	Annual Budget PPRs		
		Annual Specification (%)	No Detail (%)	None (%)
up to 25,000 L/year	23.8	20	20	60
between 25,001 and 100,000 L/year	47.6	0	82	18
between 100,001 and 250,000 L/year	23.8	40	60	0
between 250,001 and 500,000 L/year	4.8	100	0	0
Percentage of total wineries	100	24	62	14

**Table 4:** Contingency table of the medians of each variable associated to each evaluated CCP.

	Frequencies	Always (3)	Usually (2)	Hardly Ever (1)	Never (0)	High Variability
Winemaking Steps	Variable/Critical Control Point (CCP)					
1. Harvest and grape transportation	VAR1.1 Grape inspection previous harvest in vineyards.					Y
	VAR1.2 Grape inspection during harvest in vineyards.					
	VAR1.3 Transportation time of harvest from vineyards to winery.					Y
2. Harvest reception in the winery	VAR2.1 Presence of fungicide residues and/or pesticides in grapes.					Y
	VAR2.2 Presence of mycotoxins from grape rot.					
	VAR2.3 Contamination by metals (Cadmium, Lead, Arsenic) in grapes.					Y
	VAR2.4 Contamination by plant residues, dust and/or metal elements.					
3. Pre-hatching treatments	VAR3.1 Vat cleaning to eliminate residues of microorganisms.					
	VAR3.2 No residues of cleaning and disinfection products in vats.					
4. Grapes crushing and must pumping	VAR4.1 Time that remains the must in the crusher after crushing.					Y
	VAR4.2 Cleaning of crushing equipment.					
	VAR4.3 No residues of cleaning and disinfection products in vats.					
5. Sulphited and vatted	VAR5.1 Safety and purity of the additives.					Y
	VAR5.2 No microorganisms in equipment and vats.					
6. Alcoholic fermentation, maceration, vat emptying, pressing, malolactic fermentation	VAR6.1 Concentration of ethylocarbamate in fermented must.					Y
	VAR6.2 Concentration of sulphur dioxide in fermented must.					
	VAR6.3 Purity and safety of yeasts.					Y
	VAR6.4 Temperature during fermentation.					
	VAR6.5 pH of red wine during malolactic fermentation.					
	VAR6.6 Hygiene during vat emptying/pressing operations.					
	VAR6.7 Cleaning of pressing equipment.					Y
7. Racking, clarification, and filtration	VAR7.1 Cleaning procedures for vats and racking equipment.					
	VAR7.2 Maintenance and cleaning of the facilities during racking.					
	VAR7.3 Purity and safety of agents used as clarifiers of the wine.					Y
	VAR7.4 No residues of clarifiers in the wine.					Y
	VAR7.5 No weird elements from filters in the wine.					
	VAR7.6 Hygiene during clarification and filtering operations.					
	VAR7.7 No residues of cleaning and disinfection products in vats.					
8. Cold stabilization	VAR8.1 Limit concentrations of metals (traces of As, Cu, Pb) in the wine.					Y
	VAR8.2 Additives accepted by current food legislation.					
9. Bottling and labelling	VAR9.1 Bottle cleaning procedures.					Y
	VAR9.2 Cleaning procedures of the bottling line.					
	VAR9.3 No microorganisms in the bottling line.					Y
	VAR9.4 No microorganisms in bottle cap.					Y
	VAR9.5 Correct coding of the label used on the bottles.					
	VAR9.6 Correct description of allergen information on bottle labels.					
	VAR9.7 Correct description of P.D.O. information on bottle labels.					

**Table 5:** Practical implementation of principles by type of winery, according to their annual wine production and by the total of the wineries.

Annual Wine Production	% Over Total Wineries	Principle 3			Principle 4		Principle 5		Principle 6		Principle 7		
		Critical Limits (%)			Surveillance System (%)		Corrective Measures (%)		Verification Procedure (%)		Registration and Documentation System (%)		
		No	Yes (a)	Yes (b)	No	Yes	No	Yes	No	YES	No	Yes (c)	Yes (d)
up to 25,000 L/year	25	0	25	75	0	100	25	75	25	75	0	25	75
between 25,001 and 100,000 L/year	50	12.5	37.5	50	12.5	87.5	50	50	50	50	12.5	50	37.5
between 100,001 and 250,000 L/year	25	0	50	50	0	100	0	100	0	100	0	50	50
**Percentage of** **total wineries**	**100**	**6.2**	**37.5**	**56.3**	**6.2**	**93.8**	**31.3**	**68.7**	**31.3**	**68.7**	**6.2**	**43.8**	**50**

**Table 6:** Control frequency and audit achievements by type of winery, according to their annual wine production and by the total of the wineries.

Annual Wine Production	% Over Total Wineries	Principle 6				
		Frequency (%)		Audits (%)		
		No	Yes	No	Yes (e)	Yes (f)
up to 25,000 L/year	28.6	25	75	25	25	50
between 25,001 and 100,000 L/year	42.8	33.3	66.7	50	33.3	16.7
between 100,001 y 250,000 L/year	28.6	25	75	25	25	50
**Percentage of total wineries**	**100**	**28.6**	**71.4**	**35.7**	**28.6**	**35.7**

## Data Availability

Data used in this study are available on fair request to the corresponding authors.
